# Assemblages of rhizospheric and root endospheric mycobiota and their ecological associations with functional traits of rice

**DOI:** 10.1128/mbio.02733-23

**Published:** 2024-02-06

**Authors:** Junjie Guo, Huiling Ning, Yong Li, Qicheng Xu, Qirong Shen, Ning Ling, Shiwei Guo

**Affiliations:** 1 State Key Lab of Biocontrol, School of Agriculture, Shenzhen Campus of Sun Yat-sen University, Sun Yat-sen University, Shenzhen, Guangdong, China; 2 Jiangsu Provincial Key Lab for Solid Organic Waste Utilization, Jiangsu Collaborative Innovation Center for Solid Organic Waste Resource Utilization, Nanjing Agricultural University, Nanjing, China; 3 College of Plant Science and Technology, Huazhong Agricultural University, Wuhan, Hubei, China; The University of Arizona, Tucson, Arizona, USA

**Keywords:** fungi, rhizosphere, root endosphere, community assembly, plant-microbiota association

## Abstract

The soil-root interface harbors complex fungal communities that play vital roles in the fitness of host plants. However, little is known about the assembly rules and potential functions of rhizospheric and endospheric mycobiota. A greenhouse experiment was conducted to explore the fungal communities inhabiting the rhizosphere and roots of 87 rice cultivars at the tillering stage via amplicon sequencing of the fungal internal transcribed spacer 1 region. The potential relationships between these communities and host plant functional traits were also investigated using Procrustes analysis, generalized additive model fitting, and correlation analysis. The fungal microbiota exhibited greater richness, higher diversity, and lower structural variability in the rhizosphere than in the root endosphere. Compared with the root endosphere, the rhizosphere supported a larger coabundance network, with greater connectivity and stronger cohesion. Null model-based analyses revealed that dispersal limitation was primarily responsible for rhizosphere fungal community assembly, while ecological drift was the dominant process in the root endosphere. The community composition of fungi in the rhizosphere was shown to be more related to plant functional traits, such as the root/whole plant biomass, root:shoot biomass ratio, root/shoot nitrogen (N) content, and root/shoot/whole plant N accumulation, than to that in the root endosphere. Overall, at the early stage of rice growth, diverse and complex rhizospheric fungal communities are shaped by stochastic-based processes and exhibit stronger associations with plant functional traits.

**IMPORTANCE:**

The assembly processes and functions of root-associated mycobiota are among the most fascinating yet elusive topics in microbial ecology. Our results revealed that stochastic forces (dispersal limitation or ecological drift) act on fungal community assembly in both the rice rhizosphere and root endosphere at the early stage of plant growth. In addition, high covariations between the rhizosphere fungal community compositions and plant functional trait profiles were clearly demonstrated in the present study. This work provides empirical evidence of the root-associated fungal assembly principles and ecological relationships of plant functional traits with rhizospheric and root endospheric mycobiota, thereby potentially providing novel perspectives for enhancing plant performance.

## INTRODUCTION

In nature, plants coexist with a variety of microorganisms, such as bacteria, fungi, archaea, protists, and viruses (collectively termed the plant microbiota), which coevolve with their host plants and form a coherent biological entity referred to as a holobiont (1–3). Plants represent an ideal, resource-rich ecological niche that allows microbial associates to thrive; additionally, many members of the associated microbiota undoubtedly confer fitness advantages to host plants, including nutrient acquisition and uptake, disease resistance, and abiotic/biotic stress tolerance (1, 2, 4, 5). Among the plant-associated microbiota, fungi (also called mycobiota) are dominant and play key roles in plant functioning and health as mutualists, saprotrophs, or pathogens (4, 6–8). For instance, mycorrhizal fungi improve nutritional conditions and enhance the stress resistance of host plants (9–11). Some free-living saprotrophic fungi can establish facultative biotrophic interactions with plants that facilitate plant nutrient uptake (12). Therefore, understanding the community assembly rules of mycobiota and their ecological coassociations with host plants is a crucial topic for research on plant holobionts.

Most attention concerning plant holobiont research has been dedicated to the assemblage of bacterial communities at the soil-root interface (13–18), as plant roots represent the primary site for signal transduction and communication between host plants and their associated microbiota (19). Along the soil-root continuum, plant roots assemble microbiota from the soil microbial species pool into three separate microhabitats: the rhizosphere (microorganisms surrounding the root), the rhizoplane (root epiphytic microorganisms), and the root endosphere (microorganisms living inside the root) (1, 19, 20). Great progress has been made in deciphering the characterization and assembly mechanisms of rhizospheric and root endospheric microbiota over the last few years (19, 21). According to the scholarly consensus, a clear differentiation of bacteria exists between rhizosphere and root endosphere microhabitats (14–16). The bacterial microbiota switches from dense and diverse rhizosphere communities to root endosphere communities with less complexity and reduced diversity (17, 22, 23). Moreover, advances in recent years have provided quantitative evidence that bacterial microbiota establishment in the rhizosphere/endosphere is not completely stochastic (e.g., random dispersal and drift events) but rather predominantly driven by deterministic (e.g., selection) processes (17, 24–26). However, in contrast to the existing knowledge concerning bacterial community assembly at the rhizosphere-root endosphere barrier, the relative contribution of multiple ecological processes that govern the assemblage of rhizospheric and root endospheric mycobiota is still under debate (27–31).

Plant functional traits (including morphological, physiological, and phenological features) represent strategies related to plant growth, reproduction, and survival to some extent (32). A growing awareness has emerged that complex and intimate linkages exist between host plants and their associated mycobiota; these patterns of codependent plant-fungal associations are considered to be profoundly interwoven with plant functional traits (33, 34). Host-specific changes in the abundance and diversity of fungi, especially particular functional guilds, are important predictors in the predictive frameworks of observed plant trait-fungal relationships (34–36). For example, a greater degree to which a plant may depend on mycorrhizal symbioses for nutrient acquisition and uptake indicates a lower specific root length and thicker root diameter (34, 36). Plant species with higher shoot nitrogen (N) concentrations and finer roots tend to recruit fewer mycorrhizal fungi and attract diverse fungal pathogens and specialist saprotrophs (35). Thus, fungi are considered vital components of microbial-mediated mechanisms underlying plant functional traits (37). However, the understanding of the ecological linkages between rhizospheric and root endospheric mycobiota and plant functional traits from the perspective of overall fungal community composition remains limited.

Rice (*Oryza sativa* L.) is cultivated globally and consumed by more than half of the world’s population. Here, rice was selected as a model plant for investigating host-microbiota associations. The original analysis of both the rhizosphere and root endosphere fungal microbiota was performed for 87 rice varieties (Table S1) under controlled greenhouse conditions to explore the community assemblages and associated assembly processes, and these data were combined with those for rice phenotypic characteristics to evaluate the linkages between rhizospheric and root endospheric fungal community assemblages and plant functional traits. We hypothesized that, similar to bacteria (17, 24), the assemblage of rhizospheric and root endospheric fungal communities could be strongly affected by rhizospheric compartmentalization and dominated by deterministic assembly processes. Moreover, we postulated that the rhizosphere rather than the root endosphere fungal microbiota is strongly associated with plant functional trait profiles because microbial-plant interactions usually occur in the rhizosphere (3, 38). This work helps to characterize plant-microbiota associations and lays a foundation for engineering beneficial plant microbiomes for sustainable agricultural production.

## RESULTS

### Fungal diversity and co-occurrence patterns in the rhizosphere and root endosphere

**Fig 1:**
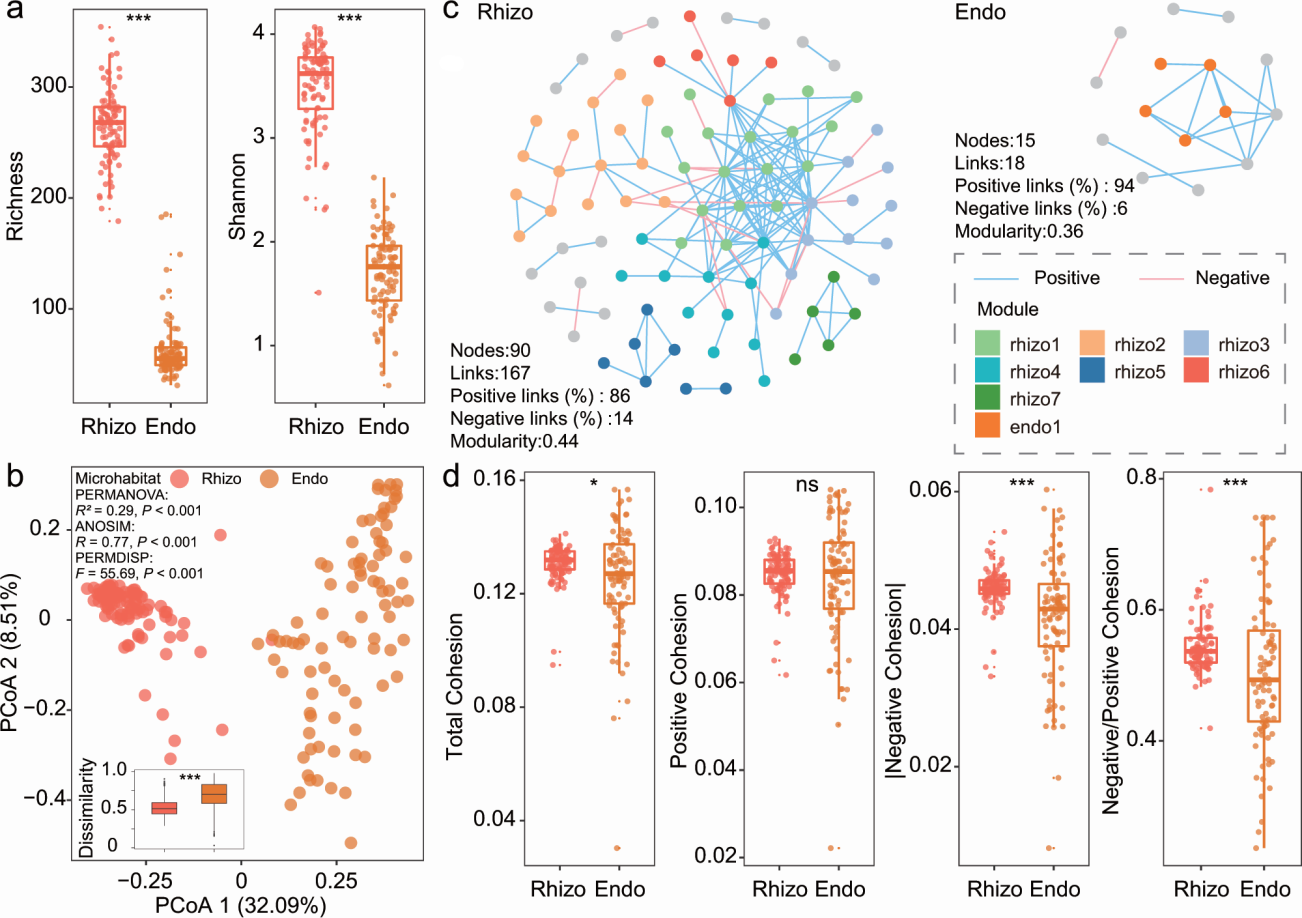
The α diversity, β diversity, and coabundance patterns of fungal communities inhabiting the rhizosphere (Rhizo) and root endosphere (Endo). (**a**) Richness and Shannon indices of fungal communities in the rhizosphere and endosphere. Statistically significant differences in α diversity between the two rhizocompartments are determined by the Wilcoxon rank sum test. (**b**) PCoA plot depicting the β diversity patterns of rhizospheric and root endospheric fungal communities based on Bray‒Curtis distances. Comparisons of fungal communities between two rhizocompartments are conducted using PERMANOVA, ANOSIM, and PERMDISP tests. Community dissimilarity is computed based on the Bray‒Curtis distance within each rhizocompartment. Statistically significant differences in dissimilarity between the two rhizocompartments are determined by the Wilcoxon rank sum test. (**c**) Coabundance networks of fungal communities in the rhizosphere and endosphere. Nodes represent the OTUs, links indicate significant correlations between the nodes, and modularity refers to the extent to which a network is divided into modules. Modules are randomly colored (nodes are colored gray when modules have fewer than five nodes). (**d**) Total cohesion, positive cohesion, absolute value of negative cohesion, and ratio of negative:positive cohesion for rhizosphere and endosphere fungal communities. Statistically significant differences in cohesion metrics between the two rhizocompartments are determined by the Wilcoxon rank sum test. The significance levels of the Wilcoxon rank sum test are as follows: *P < 0.05; **P < 0.01; ***P < 0.001; ns, not significant.

After the bioinformatic analysis pipeline (39, 40), a total of 1,492 and 624 fungal operational taxonomic units (OTUs) were identified in the rhizosphere and endosphere, respectively. Measures of within-sample diversity (α diversity) revealed a significant difference between the rhizosphere and root endosphere (*P* < 0.001) (Fig. 1a). The rhizosphere exhibited greater richness and higher Shannon index values than did the root endosphere (Fig. 1a). A principal coordinate analysis (PCoA) plot revealed that the fungal microbiota of the rhizosphere and root endosphere formed two distinct clusters that separated along the first coordinate axis (explaining 32.09% of the overall variation), suggesting an unambiguous spatial compartmentalization of the fungal microbiota [permutational multivariate analysis of variance (PERMANOVA): *R^2^* = 0.29, *P* < 0.001; similarity analysis (ANOSIM): *R* = 0.77, *P* < 0.001; Fig. 1b]. However, the significant output of the betadisper test in the taxonomic profile [permutation multivariate dispersion (PERMDISP): *F* = 55.69, *P* < 0.001] suggested that differences in taxonomic β diversity may also be an artifact of within-group dispersion (Fig. 1b). A comparison of the average community dissimilarity between the two rhizocompartments revealed that the taxonomic variability of the rhizosphere fungal microbiota was much lower than that of the root endosphere (Fig. 1b). Multiple network topological parameters consistently showed that the fungal co-occurrence patterns in the rhizosphere differed profoundly from those in the root endosphere (Fig. 1c and d). The fungal community in the rhizosphere formed a highly connected network with a larger size (nodes = 90), greater connectivity (links = 167), and stronger modular structure (modularity = 0.44) than that observed in the root endosphere (Fig. 1c). The observed potential interactions at two rhizocompartments were predominantly positive, accounting for 82%–94% of the total linkages (Fig. 1c). The proportion of negative correlations in the rhizosphere network was obviously greater than that in the endosphere network (Fig. 1c). Higher values were observed for the rhizosphere fungal community than for the root endosphere fungal community in terms of total cohesion, negative cohesion, and the ratio of negative:positive cohesion (Fig. 1d). Conversely, positive cohesion did not significantly differ between the rhizosphere and root endosphere.

### Fungal taxonomic composition and functional composition in the rhizosphere and root endosphere

**Fig 2:**
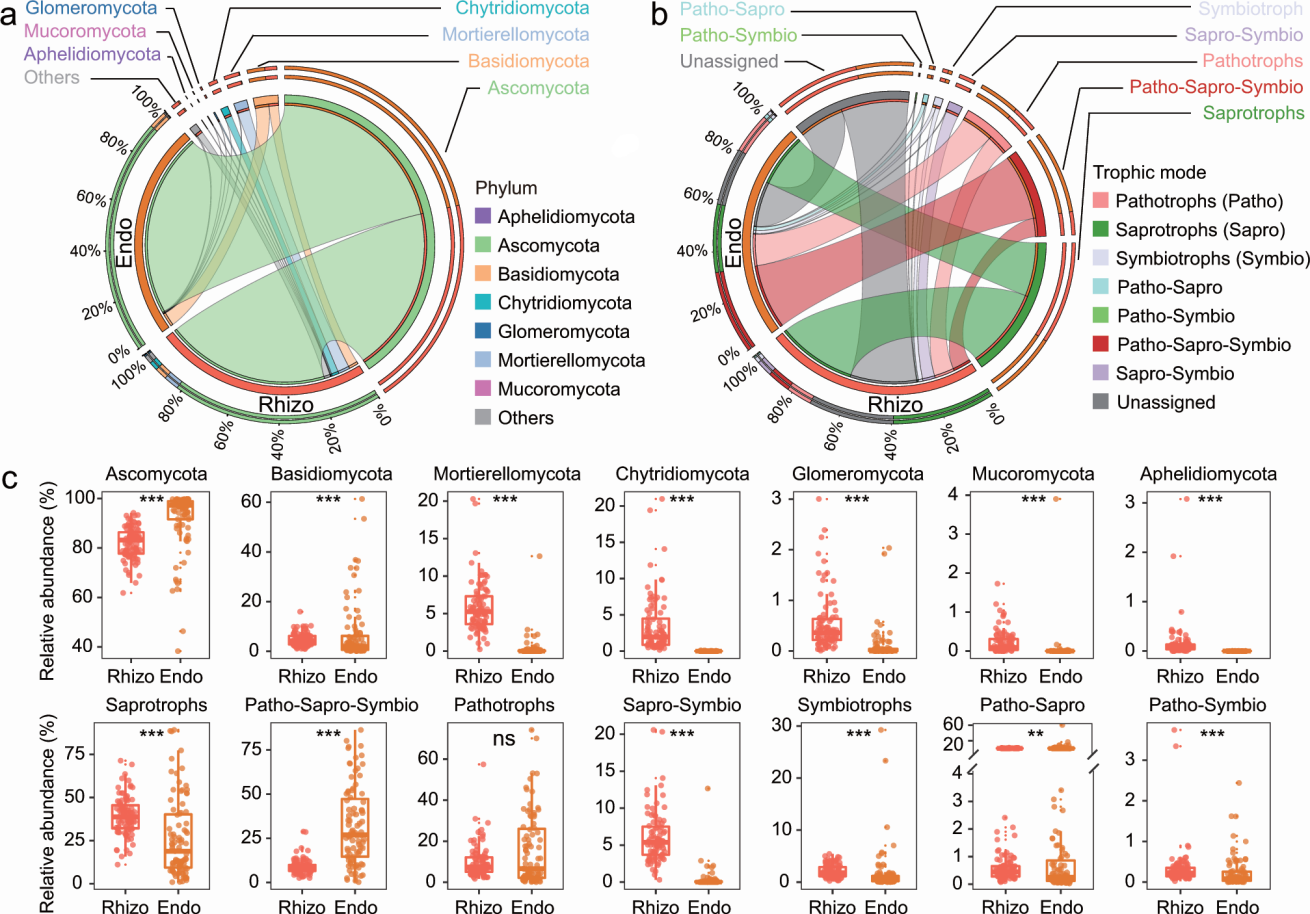
Taxonomic and functional compositions of the fungal communities inhabiting the rhizosphere (Rhizo) and root endosphere (Endo). (**a**) Relative abundance of fungal taxa in the rhizosphere and endosphere at the phylum level. (**b**) Relative abundance of fungal trophic modes between the rhizosphere and endosphere. (**c**) Comparisons of the relative abundances of major fungal phyla or trophic modes between the rhizosphere and endosphere. The left lower part of the circle represents either (**a**) the taxonomic composition or (**b**) the functional composition of the fungi inhabiting each rhizocompartment. Different colors indicate distinct fungal phyla or trophic modes, and the length represents the relative abundance of each phylum or trophic mode within each rhizocompartment (the percentage shown in the circle). The right upper part of the circle represents the distribution proportion of a certain fungal (**a**) phylum or (**b**) trophic mode in both rhizocompartments. Different colors indicate distinct rhizocompartments, and the length represents the overall distribution proportion of a certain species in both rhizocompartments. One end of the colored strip inside the circle connects to the rhizocompartment (left lower semicircle), and the other end connects to the fungal phylum or trophic mode (right upper semicircle). Statistically significant differences in relative abundance between the two rhizocompartments are determined by the Wilcoxon rank sum test. The significance levels of the Wilcoxon rank sum test are as follows: **P < 0.01; ***P < 0.001; ns, not significant.

At the phylum level, the rhizospheric and root endospheric fungal communities predominantly consisted of the phylum *Ascomycota*, which accounted for an average relative abundance of 87.07% of the fungal sequences, followed by *Basidiomycota* (5.77%), *Mortierellomycota* (3.09%), *Chytridiomycota* (1.71%), and *Glomeromycota* (0.32%) (Fig. 2a). In terms of the fungal trophic mode, saprotrophs (32.76%) and, to a lesser extent, pathotroph-saprotroph-symbiotrophs (20.53%), pathotrophs (12.78%), and saprotroph-symbiotrophs (3.21%) dominated the rhizospheric and root endospheric fungal assemblages (Fig. 2b). The phyla *Basidiomycota*, *Mortierellomycota*, *Chytridiomycota*, *Glomeromycota*, *Mucoromycota*, and *Aphelidiomycota* were present at higher relative abundance levels in the rhizosphere than in the root endosphere. Conversely, the relative abundance of the phylum *Ascomycota* was increased in the root endosphere (Fig. 2c). The rhizosphere hosted a fungal community with richer trophic modes related to saprotrophs, saprotroph-symbiotrophs, symbiotrophs, pathotroph-saprotrophs, and pathotroph-symbiotrophs. In contrast, the trophic modes of root endosphere-inhabiting fungi increased in association with pathotroph-saprotrophs-symbiotrophs compared to those in the rhizosphere (Fig. 2c).

### Eco-evolutionary processes controlling rhizospheric and root endospheric fungal communities

**Fig 3:**
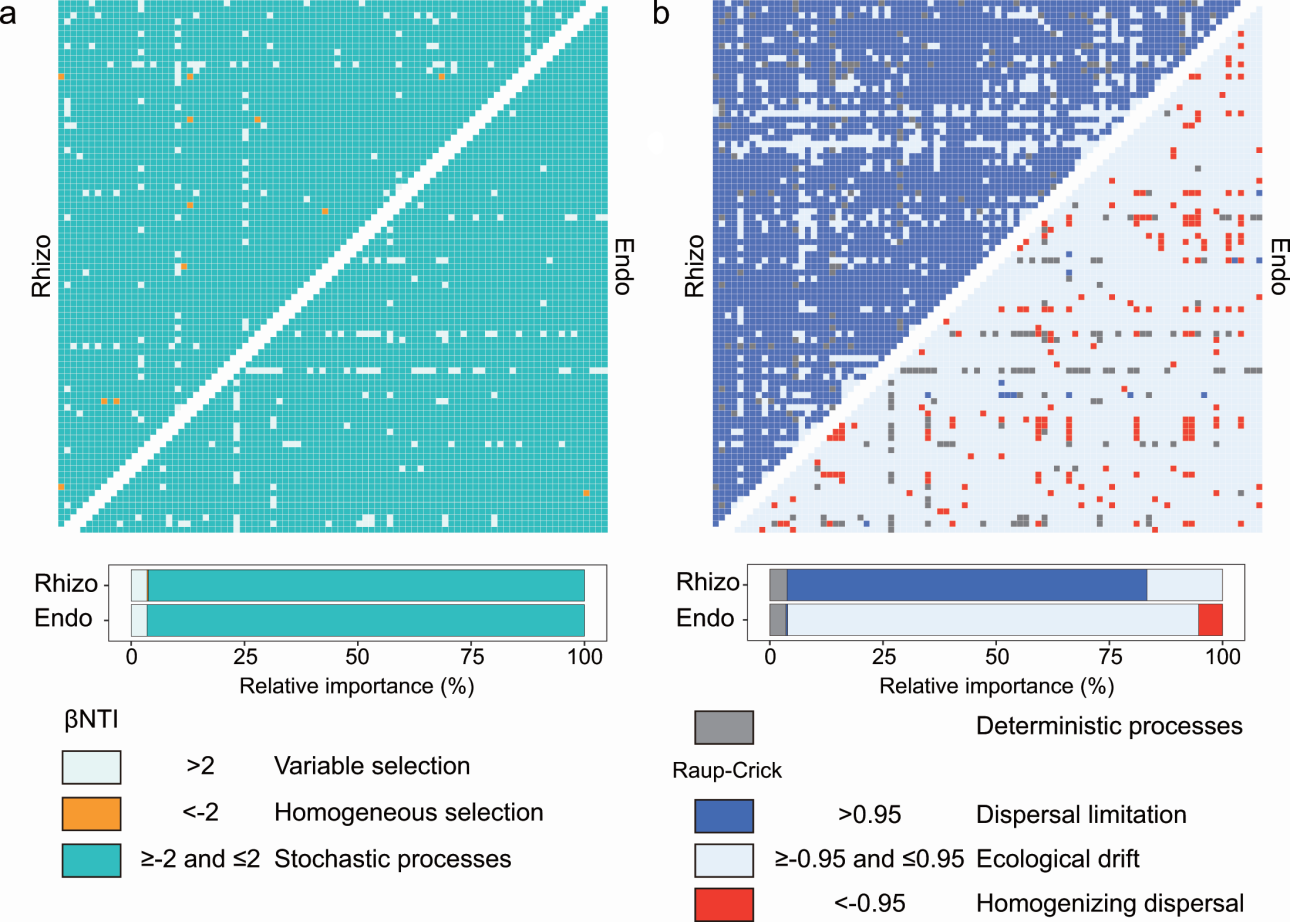
The relative importance of ecological processes that determine fungal community assembly in the rhizosphere (Rhizo) and root endosphere (Endo). (**a**) Heatmap representing the βNTI values within each rhizocompartment. The azure indicates βNTI > 2 (variable selection), the orange indicates βNTI < −2 (homogeneous selection), and the dark turquoise indicates |βNTI| < 2 (stochastic processes). The percentages depicted in horizontal stacked bar charts represent the proportions at which variable selection, homogeneous selection, and stochastic processes contribute to fungal community assembly. (**b**) Heatmap representing the Raup–Crick (Bray–Curtis) values within each rhizocompartment. The gray represents |βNTI| > 2 (deterministic processes); therefore, Raup–Crick values do not need to be considered. The royal blue indicates Raup–Crick >0.95 (dispersal limitation), the Alice blue indicates |Raup–Crick| <0.95 (ecological drift), and red indicates Raup–Crick <−0.95 (homogenizing dispersal). The percentages depicted in horizontal stacked bar charts represent the proportions in which deterministic processes, dispersal limitation, ecological drift, and homogenizing dispersal contribute to fungal community assembly.

Ecological modeling with weighted beta nearest taxon index (βNTI) and Bray‒Curtis-based Raup‒Crick (RC_Bray_) models was performed to investigate the dominant eco-evolutionary processes that governed fungal community assembly in the rhizosphere and root endosphere. Stochastic processes were revealed to play a primary role in community assembly across the fungal communities in the rhizosphere and root endosphere (Fig. 3a). Within-compartment comparisons indicated that the fungal microbiota in the rhizosphere was dominated by dispersal limitation (79.50%), while the communities in the root endosphere were subjected to ecological drift (90.78%) (Fig. 3b).

### Relationships between rhizospheric and root endospheric fungal assemblages and plant functional traits

**Fig 4:**
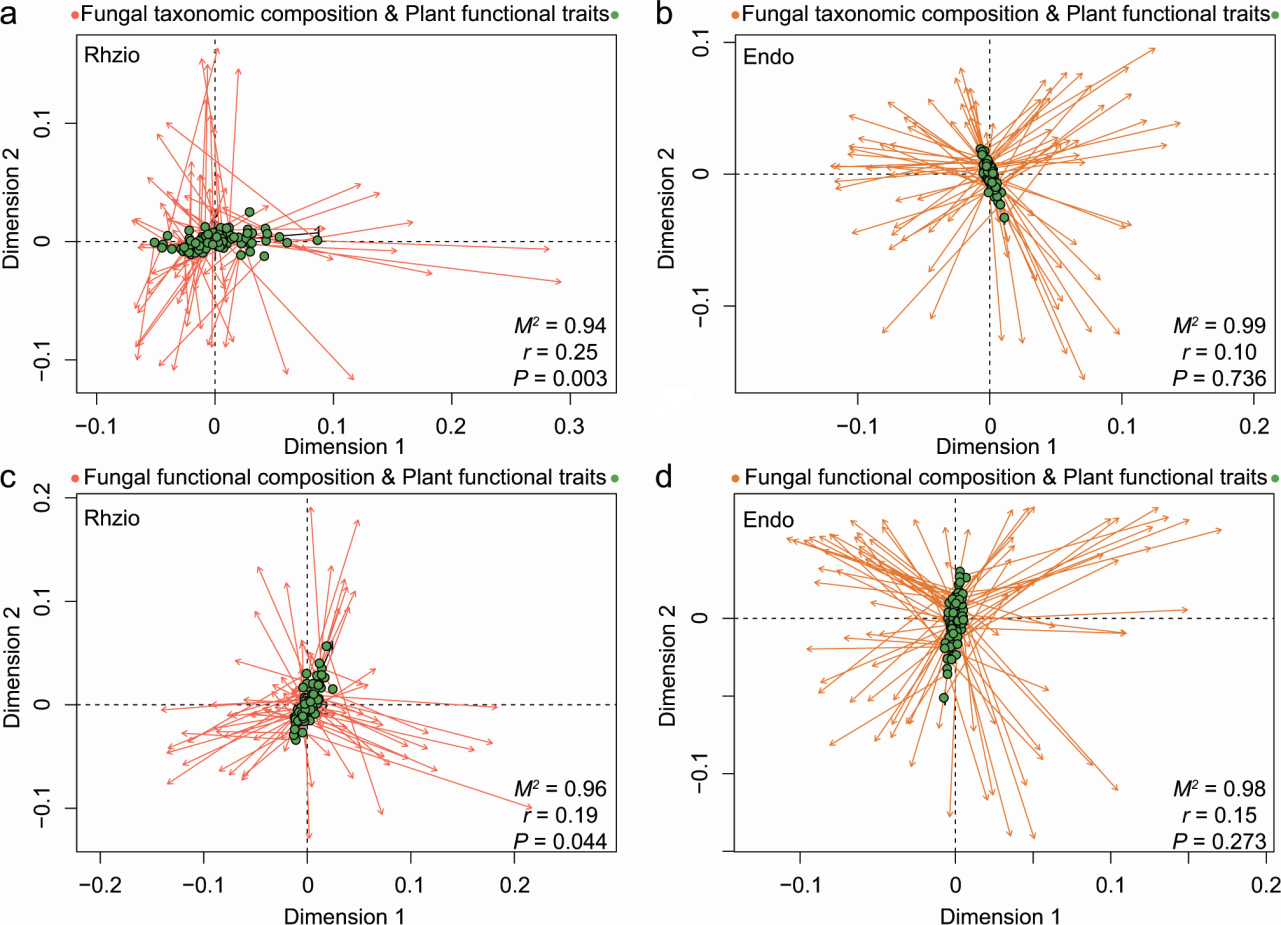
Procrustes correlations between rhizospheric and root endospheric fungal taxonomic/functional compositions and plant functional trait profiles. (**a and b**) Procrustes analysis of the rhizosphere (Rhizo) and endosphere (Endo) fungal taxonomic compositions and plant functional trait profiles. (**c and d**) Procrustes analysis of the rhizosphere (Rhizo) and endosphere (Endo) fungal functional compositions and plant functional trait profiles. *M^2^* indicates the sum of the squared distances between matched sample pairs. *r* represents the correlation in a symmetric Procrustes rotation; the *P* value is determined from 999 labeled permutations. Arrows indicate in which direction the ordination is stretched to fit the ordination of the plant functional traits to the ordination of fungal compositions.

**Fig 5:**
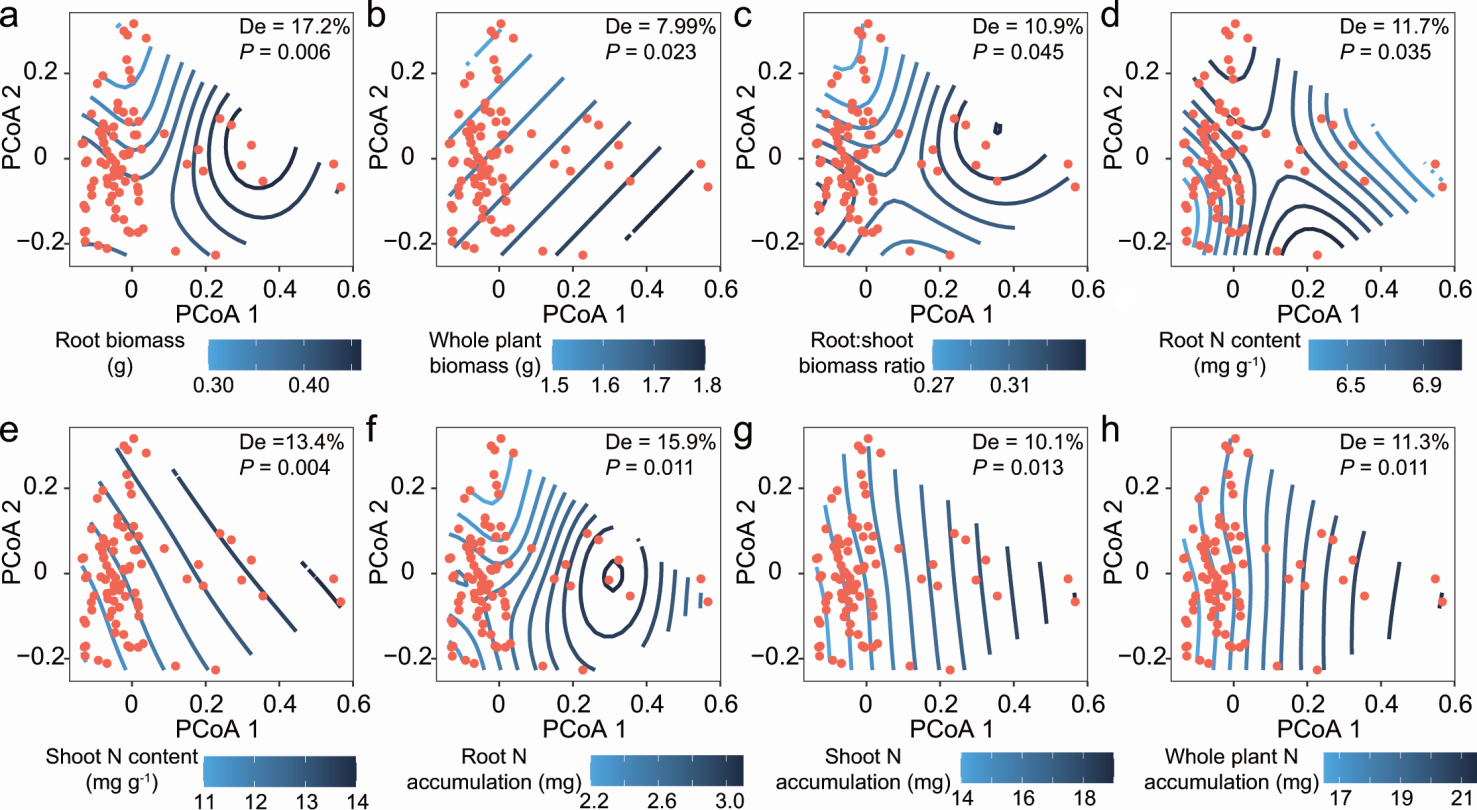
Relationships between rhizosphere fungal taxonomic composition (ordination conducted using PCoA) and specific plant functional traits. Generalized additive model (GAM) fittings illustrate significant relationships between rhizosphere fungal taxonomic composition and (**a**) root biomass, (**b**) whole plant biomass, (**c**) root:shoot biomass ratio, (**d**) root N content, (**e**) shoot N content, (**f**) root N accumulation, (**g**) shoot N accumulation, and (**h**) whole plant N accumulation. Contour lines represent plant functional trait gradients fit computed with the GAM and overlaid in the ordination space. The blue splines show the fits of the plant functional trait data from low values (light blue) to high values (dark blue) throughout the ordination. Note that the curved gradient splines indicate a nonlinear relationship between the plant trait and the fungal community composition. The gradient splines would be parallel if there is a linear relationship between the plant trait and the fungal community. De represents the deviance explained by the GAM.

**Fig 6:**
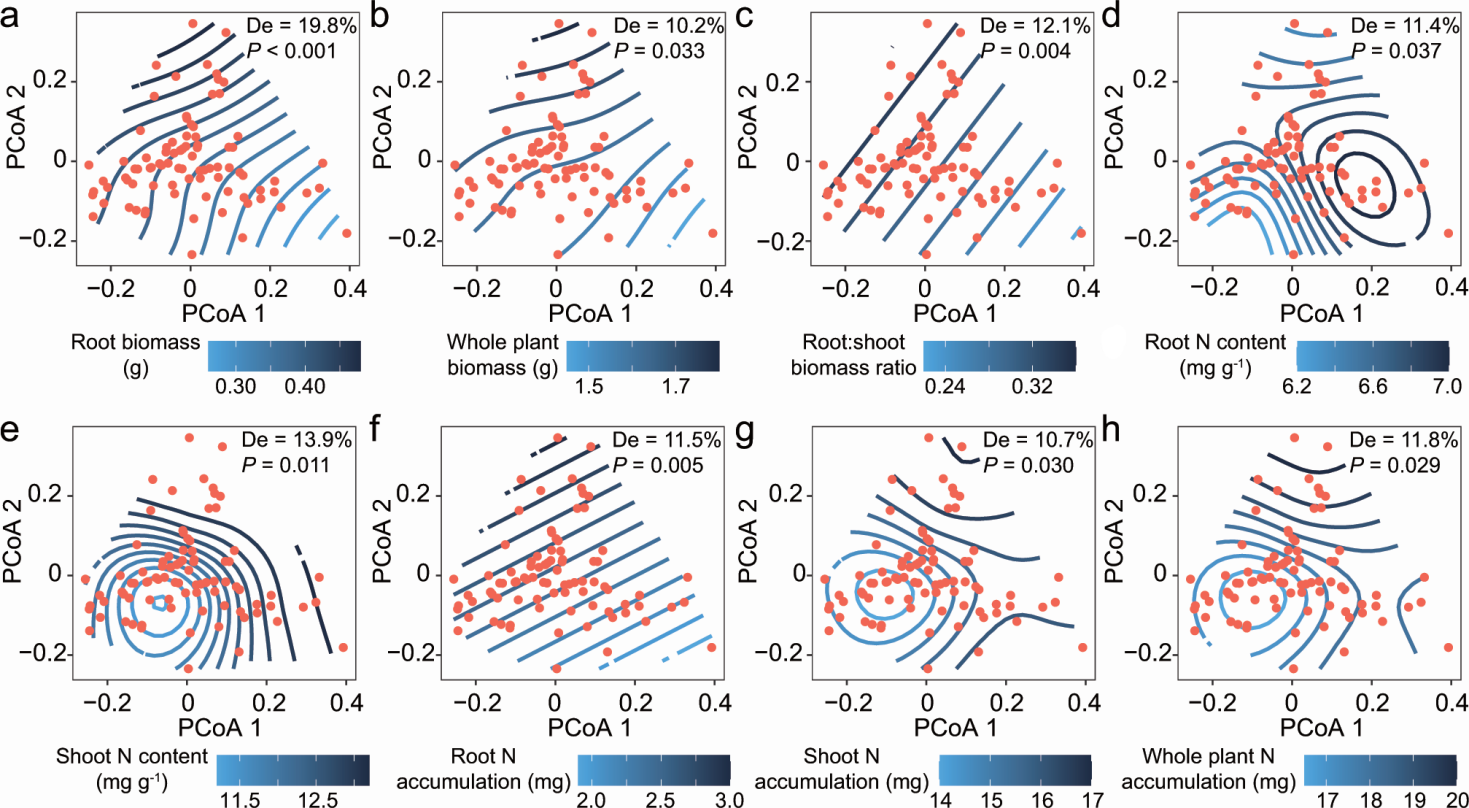
Relationships between rhizosphere fungal functional composition (ordination conducted using PCoA) and specific plant functional traits. Generalized additive model (GAM) fittings illustrate significant relationships between rhizosphere fungal functional composition and (**a**) root biomass, (**b**) whole plant biomass, (**c**) root:shoot biomass ratio, (**d**) root N content, (**e**) shoot N content, (**f**) root N accumulation, (**g**) shoot N accumulation, and (**h**) whole plant N accumulation. Contour lines represent plant functional trait gradients fit computed with the GAM and overlaid in the ordination space. The blue splines show the fits of the plant functional trait data from low values (light blue) to high values (dark blue) throughout the ordination. Note that the curved gradient splines indicate a nonlinear relationship between the plant trait and the fungal community composition. The gradient splines would be parallel if there is a linear relationship between the plant trait and the fungal community. De represents the deviance explained by the GAM.

By comparing the PCoA results of fungal taxonomic/functional composition in the rhizosphere and root endosphere with the PCoA results of plant functional trait profiles via Procrustes analysis, we found greater congruence between rhizosphere fungal composition (taxonomic: *M^2^* = 0.94, *r* = 0.25, *P* = 0.003; functional: *M^2^* = 0.96, *r* = 0.19, *P* = 0.044) and plant functional trait profiles than between root endosphere fungal composition (taxonomic: *M^2^* = 0.99, *r* = 0.10, *P* = 0.736; functional: *M^2^* = 0.98, *r* = 0.15, *P* = 0273) and plant functional trait profiles (Fig. 4). Mantel tests also confirmed that there was a strong relationship between plant functional trait dissimilarity and rhizosphere fungal taxonomic composition dissimilarity (Mantel *ρ* = 0.10, *P* = 0.042) (Fig. S1). A similar trend was observed between plant functional trait dissimilarity and rhizosphere fungal functional composition dissimilarity (Mantel *ρ* = 0.14, *P* = 0.007) (Fig. S1). To reveal potential relationships, generalized additive models were constructed to depict the relationships observed between the specific plant functional traits and the observed patterns of fungal community clustering (Tables S2 and S3). The relationships of most of the measured plant functional traits with the rhizosphere fungal taxonomic/functional compositions were significant (*P* < 0.05), except for shoot biomass (Fig. 5 and 6). The root biomass (17.2%), root N accumulation (15.9%), and shoot N content (13.4%) explained the large part of the variability in the rhizosphere taxonomic composition (Fig. 5). Similarly, the rhizosphere functional composition was explained most by the root biomass (19.8%), shoot N content (13.9%) and root:shoot biomass ratio (12.1%) (Fig. 6). However, there was a general lack of obvious relationships between the specific plant functional traits and the taxonomic/functional composition of fungi inhabiting the root endosphere (Tables S2 and S3). Only the root N content was significantly correlated with the root endosphere fungal functional profile (Fig. S2). Similarly, the rhizosphere fungal communities exhibited more fungal phyla or trophic modes related to individual plant functional trait than did the endosphere (Fig. S3).

Two approaches (network hub identification and indicator species analysis) were employed to identify the specific OTUs that potentially underlie the observed relationships between plant traits and the composition of fungal communities. Network hub OTUs exhibiting high degree and betweenness centrality were identified (Fig. S4). Fungal OTU29 (*Mortierella rishikesha* of *Mortierellomycota*, a saprotroph-symbiotroph) and OTU46 (*Ustilaginoidea virens* of *Ascomycota*, a pathotroph) were found to be hubs of the rhizosphere fungal network. The fungal network of the root endosphere, in contrast, contained only one hub OTU (OTU204), which belonged to *Thermomyces lanuginosus* of *Ascomycota*, a pathotroph-symbiotroph. A total of 129 and 13 significantly enriched OTUs, which were defined as indicator fungal OTUs, occurred in the rhizosphere and root endosphere, respectively (Fig. S5a and b). These identifiable indicator OTUs belonged primarily to the phyla *Ascomycota*, *Chytridiomycota*, *Basidiomycota*, and *Mortierellomycota*. Approximately 57% of the indicator OTUs were assigned to a trophic guild, most of which were saprotrophs. Among the fungus-specific species (including network hubs and indicators), only 46 OTUs were significantly associated with one or more measured plant functional traits. These 46 fungal OTUs exhibited strong and significant associations, primarily with the root/shoot N content and root/shoot/whole plant N accumulation, while displaying weak correlations with the root/shoot/whole plant biomass and the root:shoot biomass ratio (Fig. S5c).

## DISCUSSION

### Distinct fungal assemblages in the rice rhizosphere and root endosphere

In agreement with the findings of previous studies on other plant species (25, 41–43), a significant decrease in fungal richness and the Shannon index from the rhizosphere to the root endosphere compartment was observed in the present study (Fig. 1a), which indicated strong filtration for the recruitment of particular microorganisms at the rice rhizosphere-root endosphere barrier (1). Rhizo-compartmentalization was also the primary driving force of rhizospheric and root endospheric fungal community separation and was used to identify dominant fungal species and functional guilds in the rhizosphere and root endosphere (Fig. 1b and 2), corroborating earlier findings that the distinction in fungal microbiota composition was highly dependent on the rhizocompartment (29, 41, 44). Moreover, greater structural variability in the fungal community was observed in the root endosphere than in the rhizosphere (Fig. 1b), as the colonization and formation of endophytic communities appear to be more variable processes (14). The differences in community assemblage between the rhizocompartments were also reflected by the fungal co-occurrence pattern (Fig. 1c and d). Compared with the root endosphere, the rhizosphere harbors a larger and more complex fungal coabundance network (Fig. 1c), suggesting greater organization of the fungal community in the rhizosphere. Community cohesion, a measure of biotic interaction strength (45), was stronger in the rhizosphere than in the root endosphere (Fig. 1d), which further supported the view that the rhizosphere contained a more cohesive and stable fungal community. The abundant resources in the rhizosphere may enhance extensive interspecies cooperation and symbiotic interactions and allow more species to maintain free-living populations (42, 46). In this case, there are more opportunities for different species to directly or indirectly interact with each other, which might be responsible for increasing network complexity (47). Moreover, ecologically similar species can coexist by partitioning resources or habitats, and species do not interact with each other. In other words, the strong and intense correlation between taxa in the rhizosphere can also be interpreted as the result of convergent microbial adaptation to microhabitats surrounding the roots (48). Conversely, the root endosphere represents a relatively separate, divided, and crowded fungal microhabitat, which not only reduces fungal community diversity but also hampers interactions among fungal members (42). In addition, the presence of more isolated species in the root endosphere indicates that only particular fungal taxa can adapt to an endophytic lifestyle and fill these specific fragmented niche spaces (1, 6).

### Stochastic processes govern the assembly of rhizospheric and root endospheric fungal microbiota at the early stage of rice growth

As conceptualized by community ecology theory, four fundamental ecological processes (i.e., selection, dispersal, diversification, and ecological drift) shape the community assembly of plant-associated microbiota (49–51). In this investigation, both stochastic and deterministic components that are embedded in four fundamental ecological processes were concurrently present and determined the establishment of rhizospheric and root endospheric fungal microbiota (Fig. 3). However, contrary to expectations, the variations in the composition of fungal communities inhabiting the rhizosphere and root endosphere were chiefly introduced by stochastic processes (Fig. 3). Studies of plant-associated fungal microbiota commonly report host and environmental dependence, suggesting a certain degree of determinism during the assembly process (29, 52); however, a high level of stochasticity was also observed (27, 28, 31, 53). The assembly processes of plant-associated mycobiota alternate between deterministic and stochastic processes as plants develop (24, 27, 28, 31). In the early stages of plant growth, fungi tend to be rare and dominated by stochastic processes (27, 31), which supports our findings. Moreover, the provision of sufficient nutrients is believed to increase the stochasticity of ecological processes, which subsequently results in the dominance of stochastic process-driven fungal assembly in the rice rhizosphere and root endosphere (31, 53). Given that all the plants belong to the same species, the low contribution of deterministic processes may also be attributed to limited sources of variable host selection. Additionally, to our knowledge, community assembly process partitioning may be influenced by primer selection (54). The finding of stochasticity could partially result from potential primer bias. The specific stochastic-based processes controlling fungal community development varied between rhizocompartments (Fig. 3b). Poor spore dispersal ability may result in dispersal limitation of the rhizosphere fungal community, despite the large numbers of spores produced by fungi (55, 56). In contrast, endophytic fungal communities inhabiting roots may be prone to ecological drift due to their low overall diversity and abundance (27, 49, 50).

### Rice rhizosphere mycobiota exhibited more associations with plant functional traits

In recent studies, the potential relationships between rhizosphere fungal community composition and host plant morphological and physiological traits have been reasonably evaluated (33, 34). Similarly, the association analysis of plant functional traits revealed that plant traits were strongly correlated with both the taxonomic and functional compositions of rhizosphere fungal microbiota (Fig. 4 to 6; Fig. S1 and S3; Tables S2 and S3). The close plant trait‒microbial relationships confirmed that the fungal microbiota inhabiting the rhizosphere is a key player in plant fitness (1). However, compared with those in the rhizosphere, the strong associations between fungal microbiota profiles and plant functional traits were weakened in the root endosphere (Fig. 4; Fig. S1 to S3; Tables S2 and S3). In general, plants invest carbon (i.e., rhizodeposits) in building a belowground niche, which strengthens plant fitness in a changing environment (46). Therefore, the high covariation between the rhizosphere fungal community and plant functional traits may be related to the size of the rhizosphere effect created by a given plant. Greater functional complementarity (i.e., greater niche complementarity) and/or redundancy among species are more likely to occur in rhizosphere communities with higher diversity than in root endosphere communities (57, 58), which results in more linkages between the rhizosphere fungal community composition and plant functional characteristics. The observed trait-fungal associations could be driven by specific species or trophic guilds of fungi (34). In the present study, most rhizosphere-specific species were classified as *Ascomycota*, *Chytridiomycota*, *Basidiomycota*, or *Mortierellomycota*; were mainly saprotrophs or pathotrophs in terms of trophic guild; and exhibited a close association with plant N content and N accumulation (Fig. S4 and S5). This implies that the specific species residing in the rhizosphere may primarily participate in nitrogen acquisition by plants, thereby influencing the interaction between plant traits and fungal communities (59). In addition, the diverse fungal species inhabiting the rhizosphere may not only necessarily harbor direct beneficial functions but also contribute to making the rhizosphere a nutrient hotspot for plants through the degradation of organic matter (46). For instance, a saprophytic fungus (i.e., OTU29) with phylogenetic associations with the genus *Mortierella* was enriched in the rhizosphere and acted as a hub node in the coabundance network (Fig. S4 and S5). The genus *Mortierella*, which is among the most abundant and diverse saprophytic fungi, has been proven to be a reliable indicator for predicting plant agronomic traits (60, 61). In contrast, most fungi live within roots as commensal endophytes; they have no apparent effects on plant performance in most cases and become functional only under specific conditions (6). We emphasize that the use of universal fungal primers to characterize the entire fungal community potentially introduces bias and results in a low relative abundance of some taxa, such as arbuscular mycorrhizal fungi (62, 63). This study is also limited by the low completeness of fungal trophic guild assignments, which poses challenges for conducting functional assessments (64). Despite these limitations, the findings presented herein provide evidence that supports the existence of substantial covariation between rhizosphere mycobiota and plant functional traits (34).

### Conclusions

Stochastic processes dominate the assembly of both rhizosphere (dispersal limitation) and root endosphere (ecological drift) fungal communities at the soil-root interface during the early stage of plant growth. The degree of association between complex and diverse rhizosphere fungal communities and plant functional traits was greater than that between the simplified root endosphere fungal community and plant functional traits. Our work goes beyond simply describing the diversity patterns of rhizospheric and root endospheric fungal communities to decipher the assembly mechanisms structuring rhizospheric and root endospheric mycobiota and to highlight the stronger links between rhizosphere fungal microbiota and plant functional traits. These results could inform future efforts to engineer beneficial root-associated microbiomes for improving plant performance.

## MATERIALS AND METHODS

### Rice growth, plant functional trait assessment, and sample collection for rhizospheric and root endospheric mycobiota profiling

To investigate a “general” fungal assemblage across different rice cultivars, a total of 87 cultivated rice varieties were selected for the greenhouse experiment (Table S1). The seeds were surface sterilized with hypochlorite solution (1.5% of active Cl) for 30 min and germinated on moist filter paper. Soil was collected from the top layer (0–20 cm) of agricultural soil situated on the outskirts of Nanjing (118°53′59″E, 32°2′13″N), Jiangsu Province, China. The soil was thoroughly homogenized after being passed through a 4-mm sieve and subsequently utilized to fill the pots. After germination, the plants were moved to a greenhouse and transplanted into soil pots (six pots per variety). The plants were grown under natural light in a greenhouse with day/night temperatures maintained at 30°C/25°C.

The pots were irrigated daily to keep the soil submerged, and nutrients were supplied to each pot weekly beginning 6 weeks after transplantation. The components of the nutrient solution were as follows: macronutrients (mmol/L), 2.86 N as (NH_4_)_2_SO_4_ and Ca(NO_3_)_2_, 0.32 P as KH_2_PO_4_, 1.02 K as K_2_SO_4_ and KH_2_PO_4_, and 1.65  Mg as MgSO_4_; micronutrients (μmol/L), 35.8 Fe as Fe-EDTA, 9.10 Mn as MnCl_2_·4H_2_O, 0.52 Mo as (NH_4_)_6_Mo_7_O_24_·4H_2_O, 18.5 B as H_3_BO_3_, 0.15 Zn as ZnSO_4_·7H_2_O, 0.16 Cu as CuSO_4_·5H_2_O, and 100 Si as Na_2_SiO_3_·9H_2_O (65). The plants were cultivated for a duration of 9 weeks and harvested at the tillering stage, which represents an early phase in the growth of rice plants. Plant functional trait assessment was carried out on half of the samples, and the rest of the samples were processed for rhizospheric and root endospheric fungal community analysis.

A suite of morphological and physiological traits of plants (i.e., plant functional traits), including root biomass, shoot biomass, whole-plant biomass, root:shoot biomass ratio, root N content, shoot N content, root N accumulation, shoot N accumulation, and whole-plant N accumulation, were examined. Plant biomass (root, shoot, and whole plant dry weight) was measured after the plants were oven dried at 105°C for 30  min and then at 70°C to a constant weight. Dried plant tissues were digested with H_2_SO_4_-H_2_O_2_ at 260°C–280°C, and the N concentrations were determined by using a continuous-flow autoanalyzer (AA3, SEAL Analytical, Norderstedt, Germany). The N accumulation of each plant component was determined by multiplying the biomass by the N concentration.

Two rhizocompartments (i.e., the rhizosphere and root endosphere compartments) were sampled following a previously described procedure (17). Briefly, the loose soil aggregates were removed by shaking the entire root. The roots were subsequently transferred to sterile Falcon tubes (50 mL) containing sterile water (15 mL). After vortexing the roots, the soil suspension was subjected to centrifugation, and the resulting pellet was collected as the rhizosphere compartment. For collection of the root endosphere compartment, the roots were further thoroughly washed in sterile water. The water-washed roots were sterilized by successively submerging the tissues in hypochlorite solution (3% of active Cl) for 2 min and in 70% ethanol for 2 min. The samples were subsequently rinsed three times with sterile distilled water to remove microorganisms from the rhizoplane. The effectiveness of the sterilization process was confirmed by inoculating 100 µL of water from the final rinse onto potato dextrose agar plates. Subsequently, the plates were scrutinized to verify the absence of microbial growth (66, 67).

### DNA extraction and fungal community analysis by amplicon sequencing

DNA was extracted from rhizosphere and root endosphere samples according to the protocol of the DNeasy PowerSoil Kit (Qiagen, Hilden, Germany). Prior to DNA extraction, the root endosphere samples were frozen in liquid nitrogen and subsequently ground for homogenization. Three replicates of rhizospheric or endospheric DNA samples collected from the same rice variety were pooled together as a representative sample (17) and stored at −80°C until further analyses. Finally, 87 rhizospheric representative DNA samples and 87 endospheric representative DNA samples were generated for internal transcribed spacer (ITS) amplicon sequencing. Given that only one rhizospheric representative sample and one root endospheric representative sample were ultimately obtained for each rice variety, the evaluation of differences among varieties was not performed.

The fungal primer set with adaptors and barcodes used for fungal ITS1 region amplification was as follows: ITS1-F (5′-CTTGGTCATTTAGAGGAAGTAA-3′) (68) and ITS2 (5′-GCTGCGTTCTTCATCGATGC-3′) (69). PCR amplification was performed in a 10-µL mixture containing 1 µL of 10× TopTaq Buffer, 0.2 µL of TopTaq DNA Polymerase (5 U µL^−1^), 0.8 µL of dNTPs (2.5 mM), 1 µL of each primer (2 µM), and 1 µL of DNA template. The thermal cycling procedure was as follows: initial denaturation at 94°C for 2 min; 28 cycles of 94°C for 20 s, 55°C for 30 s, and 72°C for 1 min; and a final extension of 2 min at 72°C. The experiment was performed with negative controls (sterile distilled water) and positive controls (with known quantities and combinations of microbiomes) throughout. Paired-end (2 × 250 bp) sequencing was performed on the HiSeq platform (Illumina, USA) with technical support from Genesky Biotechnology Co., Ltd. (Shanghai, China).

Amplicon data were processed using USEARCH v.11.0 (39). Briefly, the raw paired-end reads for each sample were merged, primer sequences were trimmed, and low-quality reads (length <190 bp, total expected errors >0.5) were removed. The remaining qualified reads were subsequently clustered into OTUs at a 97% minimum similarity threshold by using the UPARSE algorithm (40). Fungal taxonomy was assigned using the Ribosomal Database Project classifier (70) with the UNITE database version 8.3 (71). The OTU table was rarefied to 14,424 sequences (the minimum sequencing depth) for downstream analyses. Functional annotations (trophic modes and functional guilds) of fungal taxa were carried out using FUNGuild (72, 73). Analyses of both the richness and Shannon indices were performed using the alpha_div command from USEARCH v.11.0 (39).

### Network construction and cohesion statistics

Coabundance networks were established by measuring the covariations across 87 samples for each rhizocompartment. OTUs that were found in more than 20% of the samples (350 OTUs for the rhizosphere and 67 OTUs for the root endosphere) were retained for network construction to avoid spurious correlations (74). The inference of pairwise correlations and corresponding *P* values was performed using the SparCC (sparse correlations for compositional data) algorithm with 20 iterations and 100 bootstraps (75). Only robust (SparCC correlation magnitude <−0.30 or  >0.30) and statistically significant (*P* < 0.05) inferred correlations were reserved (76). Ecological modules, a cluster of nodes that are highly interconnected, were identified by using the greedy optimization of modularity algorithm (“igraph” package of R). Networks were visualized using Gephi 0.9.2-beta software (77). The network hub OTUs of each network were defined as nodes that ranked in the top 3% in terms of both degree and betweenness centrality (17, 76). In addition, cohesion statistics, a metric for quantifying the complexity and stability of microbial communities (78), were calculated within each rhizocompartment according to the developed workflow (45). Cohesion metrics were calculated as the average corrected pairwise correlations between microbial taxa (e.g., OTUs) within a sample. The workflow produces two cohesion values (i.e., positive and negative cohesion, the sum of the significant positive or negative correlations between individual taxa weighted by relative abundances) for each sample that quantify the connectivity arising from positive and negative co-occurrences between microbial taxa. Positive cohesion and negative cohesion are bounded by 0 to 1 and −1 to 0, respectively, with higher absolute values signifying more and/or stronger species interactions (45, 78). Total cohesion is the sum of the absolute values of both positive cohesion and negative cohesion. Moreover, a larger negative cohesion value indicates a more stable community (45, 78).

### Estimates of eco-evolutionary processes

The relative contributions of deterministic and stochastic processes that determine the assembly of rhizospheric and root endospheric fungal communities were estimated according to a null model-based statistical framework described previously (27, 79, 80). Briefly, a hierarchical phylogeny of fungal OTUs was generated by subsetting the fungal backbone classification tree (taxonomy_to_tree.pl script) (27, 81). The weighted βNTI, the difference between an observed beta mean nearest taxon distance value and the null-model estimate, was determined between each pair of communities (80). The combination of the βNTI value and the RC_Bray_ was used to quantify the relative contributions of deterministic (i.e., variable selection and homogeneous selection) and stochastic (i.e., dispersal limitation, homogenizing dispersal and ecological drift) processes. All null model-based analyses were performed using a Galaxy-based pipeline (http://dmap.denglab.org.cn) (82).

### Statistical analyses

Differences in α diversity, community dissimilarity, and cohesion between the rhizosphere and root endosphere were compared via the Wilcoxon rank sum test with false discovery rate (FDR) correction. The Wilcoxon rank sum test with FDR was also performed to compare the relative abundance of fungal phyla or trophic modes between the rhizosphere and root endosphere. The β diversity patterns of the fungal communities among the different samples were investigated via PCoA based on the Bray–Curtis distance. ANOSIM and PERMANOVA were conducted to analyze the effect of the rhizocompartment on fungal community dissimilarity. PERMDISP was also analyzed by using the “betadisper” function of the “vegan” package, version 2.5–6 (83). The relationships between rhizocompartment types and the relative abundances of fungal phyla or trophic modes were visualized via Circos software (http://circos.ca/). The fungal taxa indicative of a particular rhizocompartment were identified using indicator species analyses (“indval” function in the “labdsv” package version 2.0–1) (84). OTUs with indicator values ≥0.5 and *P* values <0.05 were defined as fungal indicators. The mean value of specific functional traits of each rice variety was calculated and matched with the rhizospheric and root endospheric fungal community data. Procrustes analyses were performed using PCoA plots as input on the basis of the matrix of fungal community taxonomic/functional composition (Bray‒Curtis) and plant functional traits (Euclidian) to assess the correlation between rhizosphere/endosphere fungal communities and plant traits (83). Moreover, the relationships between the rhizosphere/endosphere fungal community composition dissimilarities (Bray‒Curtis) and plant functional trait dissimilarities (Euclidian) were also estimated using the Mantel test (Spearman’s rank correlation). Generalized additive model fitting, which allows for both linear and nonlinear fits, was incorporated in the PCoA ordination (Bray‒Curtis distance) to assess the relationships between the rhizosphere/endosphere fungal community composition and specific plant functional traits, using the “ordisurf” function in the “vegan” package version 2.5–6 (83). Spearman’s rank correlation analyses were also conducted to evaluate the relationships between plant functional traits and the relative abundance of fungal phyla or trophic modes. Moreover, the relationships between plant functional traits and the relative abundance of network hub OTUs or indicator OTUs were also assessed through Spearman analyses.

## Data Availability

All the raw sequence data were submitted to the NCBI Sequence Read Archive (http://www.ncbi.nlm.nih.gov/Traces/sra/) database under accession number PRJNA760673.

## SUPPLEMENTAL MATERIAL

Supplemental figures mbio.02733-23-s0001.docx Fig. S1 to S5.

Supplemental tables mbio.02733-23-s0002.docx Table S1 to S3.

ASM does not own the copyrights to Supplemental Material that may be linked to, or accessed through, an article. The authors have granted ASM a non-exclusive, world-wide license to publish the Supplemental Material files. Please contact the corresponding author directly for reuse.
