# Supplementary material for: Assemblages of rhizospheric and root endospheric mycobiota and their ecological associations with functional traits of rice
Source: mBio. 2024 Feb 6;15(3):e02733-23. doi: 10.1128/mbio.02733-23 (PMC10936437; doi:10.1128/mbio.02733-23)
Supplement: Supplemental figures — Fig. S1 to S5. [file mbio.02733-23-s0001.docx]

Supplementary Figure Legend:

Figure S1. Relationships between root-associated fungal taxonomic/functional composition and plant functional traits profile. (a-b) Rhizosphere (Rhizo) and endosphere (Endo) fungal taxonomic composition dissimilarity (x-axis) vs. plant functional traits dissimilarity (y-axis). (c-d) Rhizosphere (Rhizo) and endosphere (Endo) fungal functional composition dissimilarity (x-axis) vs. plant functional traits dissimilarity (y-axis). The background color indicates density of datapoints (BACLs). Individual data points are not shown, except those falling in low density areas (black dots).

Figure S2. Relationship between endosphere fungal functional composition (ordination conducted using PCoA) and specific plant functional traits. Generalized additive model (GAM) fitting illustrate a significant relationship between endosphere fungal functional composition and root N content. Contour lines represent plant functional trait gradients fit computed with the GAM and overlaid in the ordination space. The blue splines show the fits of the plant functional trait data from low values (light blue) to high values (dark blue) throughout the ordination. Note that the curved gradient splines indicate a nonlinear relationship between the plant trait and the fungal community composition. The gradient splines would be parallel if there is a linear relationship between the plant trait and the fungal community. De represents the deviance explained by the GAM.

Figure S3. Spearman’s rank correlation between plant functional traits and relative abundance of major fungal phyla, as well as for relative abundances of fungal trophic modes.

Figure S4. Network hubs of rhizosphere (Rhizo) and root endosphere (Endo).

Figure S5. Indicator fungal OTUs inhabiting rhizosphere (Rhizo) or root endosphere (Endo) and associations between specific fungal OTUs and plant functional traits. (a) Taxonomic annotation of the indicator fungal OTUs inhabiting rhizosphere or root endosphere at the phylum level. Circles represent OTUs. (b) Functional annotation of the indicator fungal OTUs inhabiting rhizosphere or root endosphere at the trophic mode level. Circles represent OTUs. (c) Spearman’s rank correlation between plant functional traits and relative abundance of specific fungal OTUs. Only significant relationships are shown.


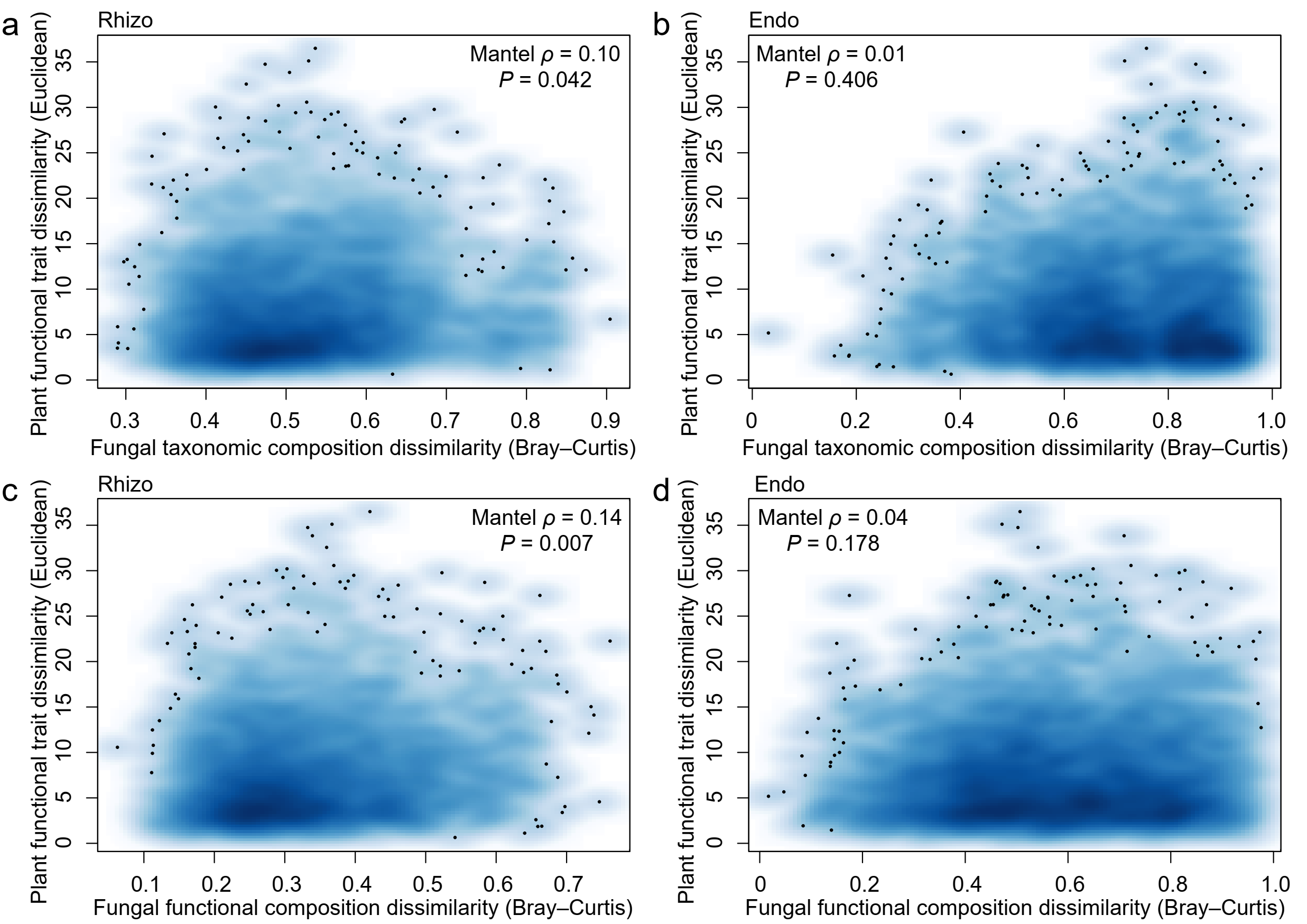


Figure S1 Relationships between root-associated fungal taxonomic/functional composition and plant functional traits profile. (a-b) Rhizosphere (Rhizo) and endosphere (Endo) fungal taxonomic composition dissimilarity (x-axis) vs. plant functional traits dissimilarity (y-axis). (c-d) Rhizosphere (Rhizo) and endosphere (Endo) fungal functional composition dissimilarity (x-axis) vs. plant functional traits dissimilarity (y-axis). The background color indicates density of datapoints (BACLs). Individual data points are not shown, except those falling in low density areas (black dots).


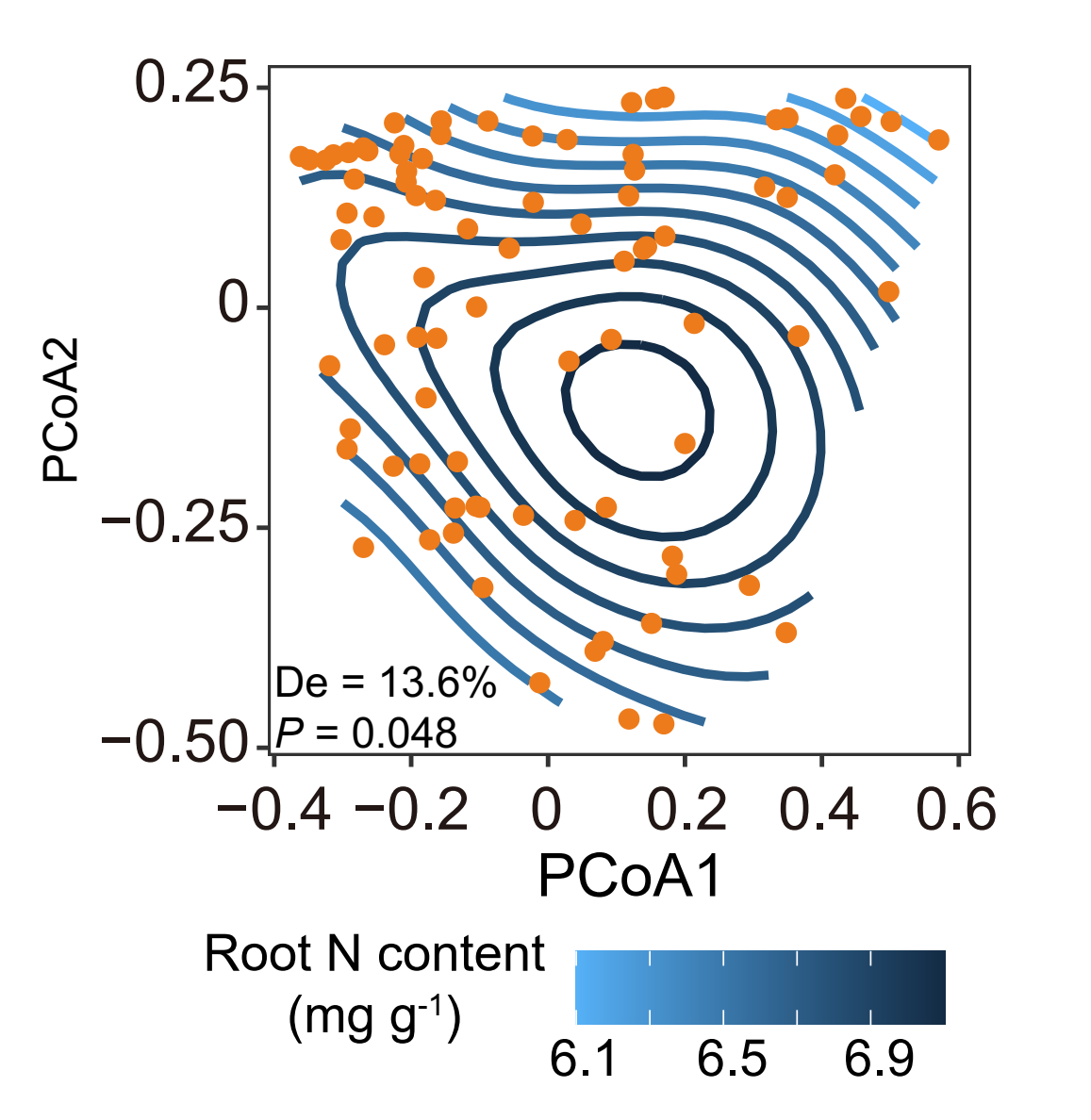


Figure S2. Relationship between endosphere fungal functional composition (ordination conducted using PCoA) and specific plant functional traits. Generalized additive model (GAM) fitting illustrate a significant relationship between endosphere fungal functional composition and root N content. Contour lines represent plant functional trait gradients fit computed with the GAM and overlaid in the ordination space. The blue splines show the fits of the plant functional trait data from low values (light blue) to high values (dark blue) throughout the ordination. Note that the curved gradient splines indicate a nonlinear relationship between the plant trait and the fungal community composition. The gradient splines would be parallel if there is a linear relationship between the plant trait and the fungal community. De represents the deviance explained by the GAM.


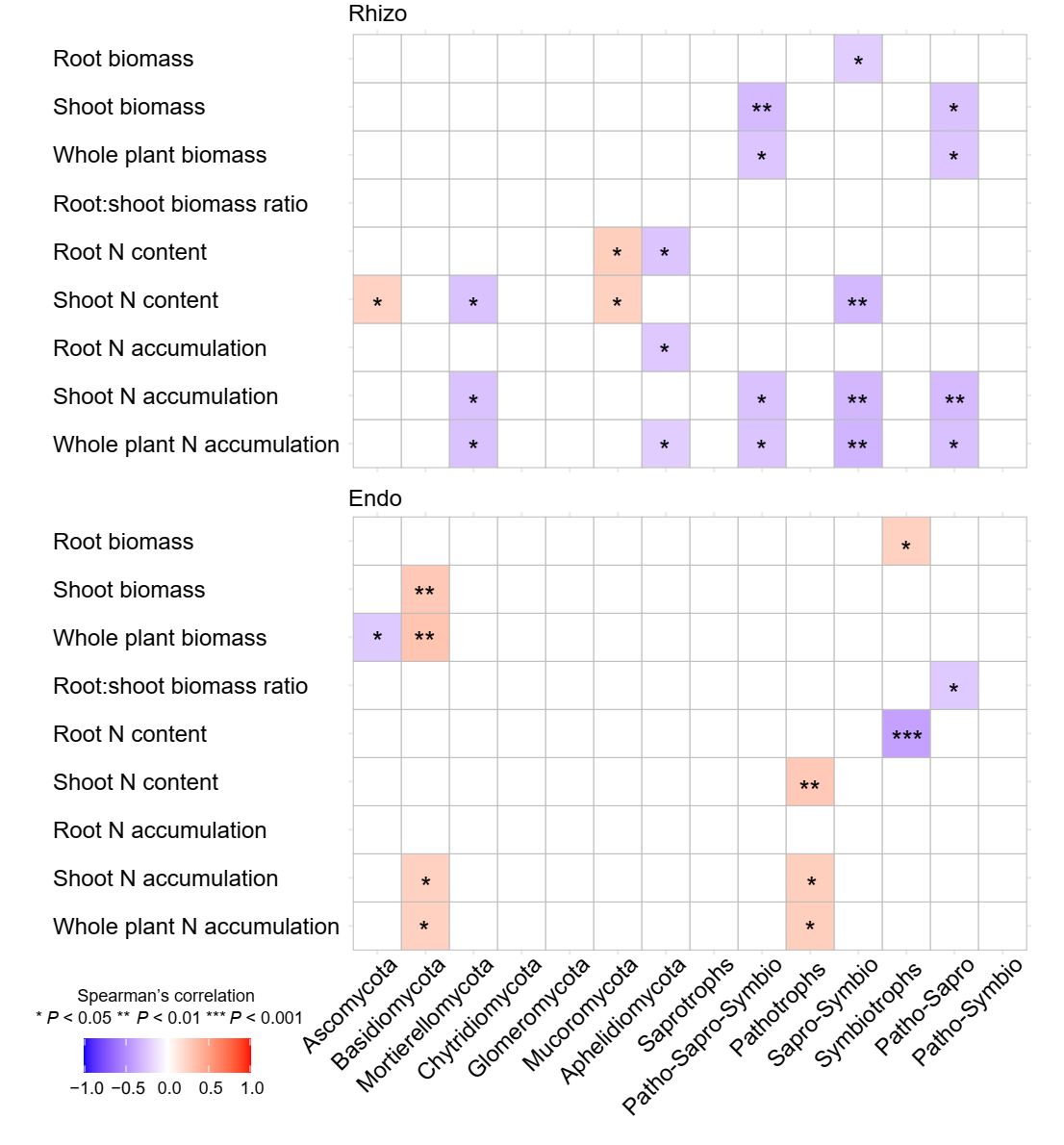


Figure S3 Spearman’s rank correlation between plant functional traits and relative abundance of major fungal phyla in rhizosphere (Rhizo) and root endosphere (Endo), as well as for relative abundances of fungal trophic modes.


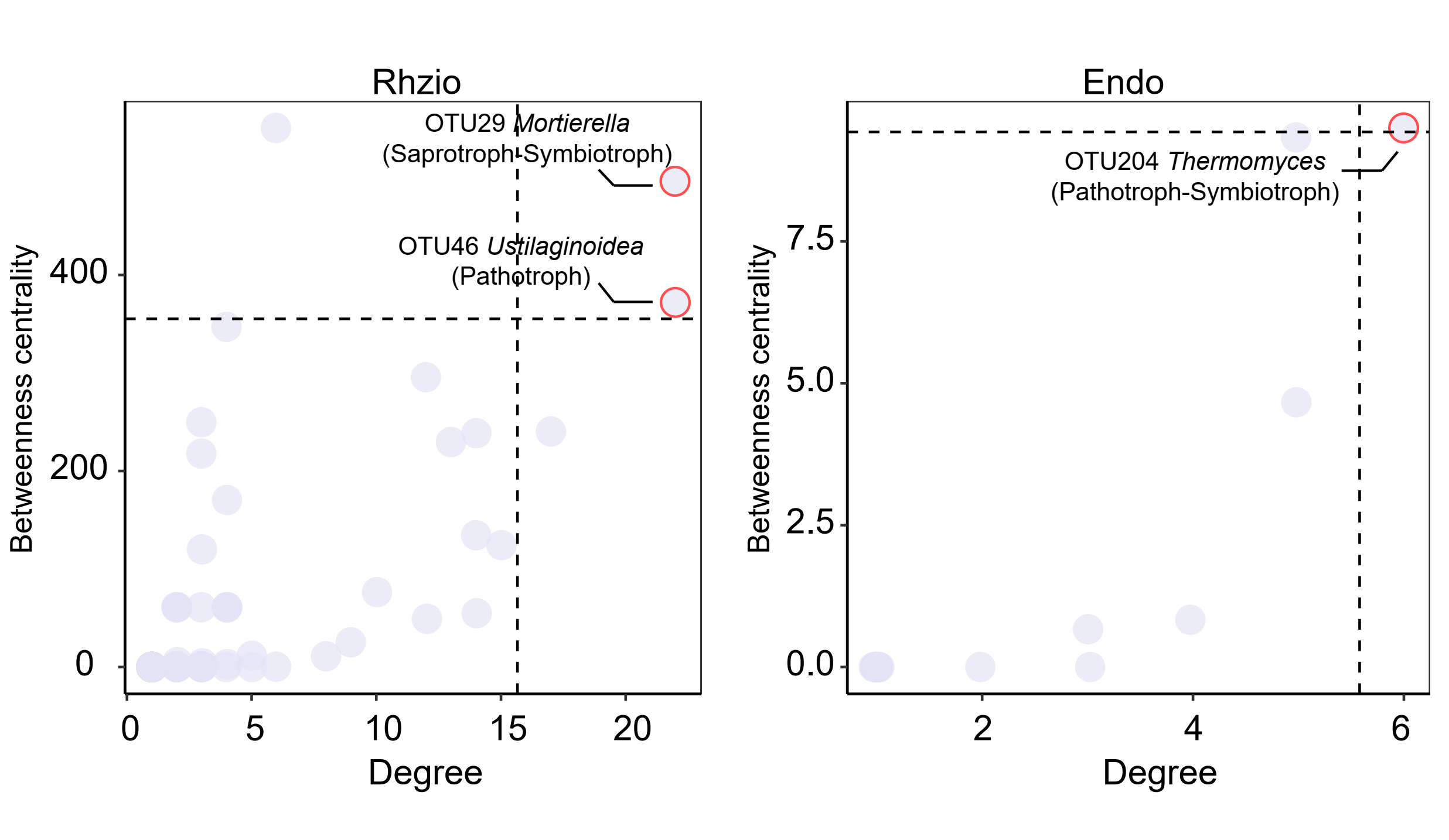


Figure S4 Network hubs of rhizosphere (Rhizo) and root endosphere (Endo).


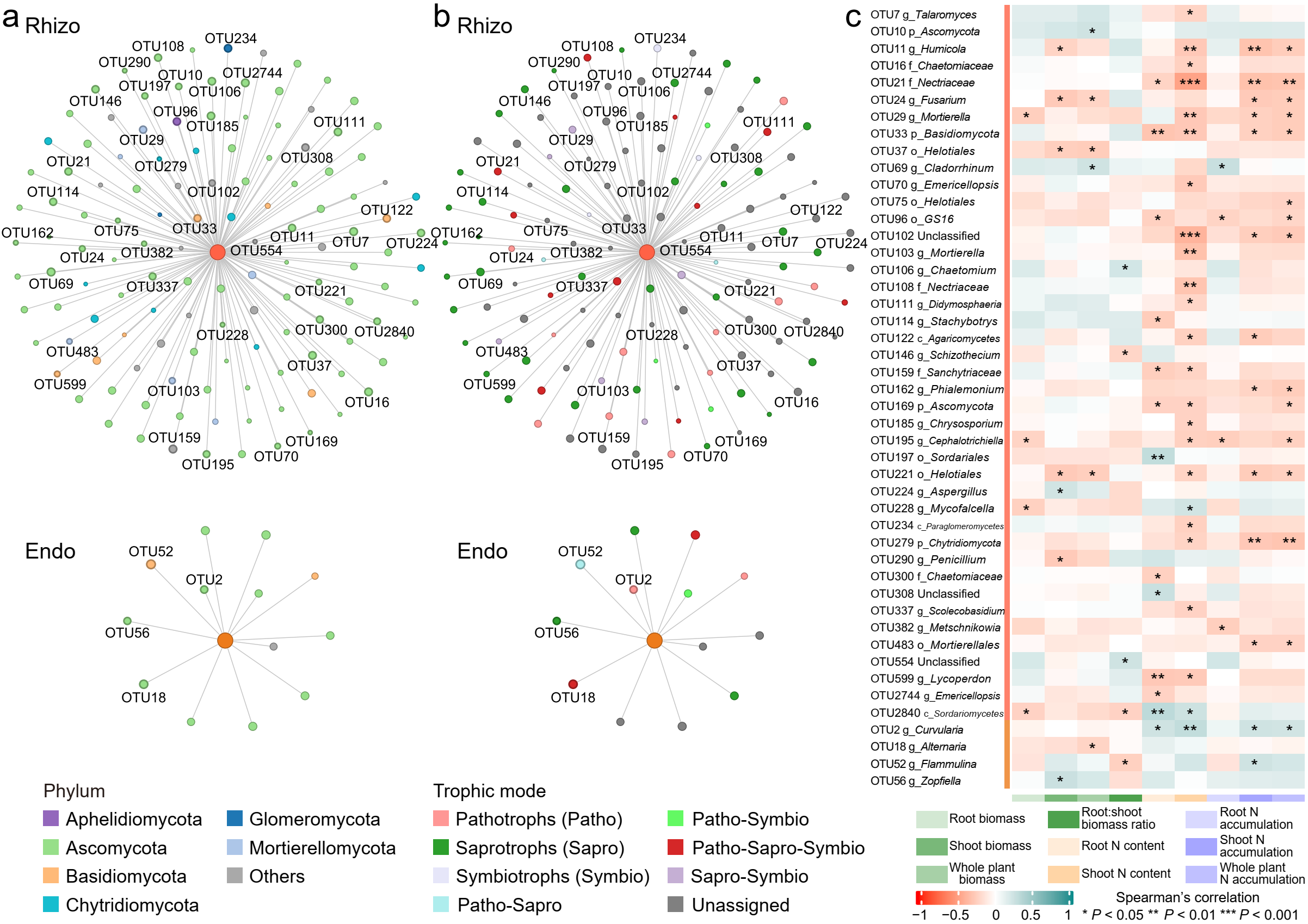


Figure S5 Indicator fungal OTUs inhabiting rhizosphere (Rhizo) or root endosphere (Endo) and associations between specific fungal OTUs and plant functional traits. (a) Taxonomic annotation of the indicator fungal OTUs inhabiting rhizosphere or root endosphere at the phylum level. Circles represent OTUs. (b) Functional annotation of the indicator fungal OTUs inhabiting rhizosphere or root endosphere at the trophic mode level. Circles represent OTUs. (c) Spearman’s rank correlation between plant functional traits and relative abundance of specific fungal OTUs. Only significant relationships are shown.
