# Supplementary material for: Assemblages of rhizospheric and root endospheric mycobiota and their ecological associations with functional traits of rice
Source: mBio. 2024 Feb 6;15(3):e02733-23. doi: 10.1128/mbio.02733-23 (PMC10936437; doi:10.1128/mbio.02733-23)
Supplement: Supplemental tables — Table S1 to S3. [file mbio.02733-23-s0002.docx]

Supplementary Tables Legend:

Table S1. Information on the cultivated rice varieties used in this study.

Table S2. Generalized additive model (GAM) analysis results (the deviance explained and the corresponding *P* values of the smoothing term) for rhizosphere fungal taxonomic composition and specific plant functional traits.

Table S3. Generalized additive model (GAM) analysis results (the deviance explained and the corresponding *P* values of the smoothing term) for rhizosphere fungal functional composition and specific plant functional traits.

Table S1. Information on the cultivated rice varieties used in this study.

| Rice variety | Origin | Subspecies |
| --- | --- | --- |
| ITALICA AGOSTANO | Poland | JAPONICA |
| LKVR | Hungary | JAPONICA |
| KOPANCSI KEREK | Hungary | JAPONICA |
| KOPANCSI RESISTA | Hungary | JAPONICA |
| BANLOC | Hungary | JAPONICA |
| PANOJA(E.E.A. 580) | Argentina | JAPONICA |
| MOCORETA F.A. | Argentina | INDICA |
| CHOKOTO | Japan | INDICA |
| B 52-2579(B61-5819) | United States | INDICA |
| CHAMPA | Iran | INDICA |
| DOM SIAH | Iran | INDICA |
| 69 C 5067 | United States | INDICA |
| CHIYOMINORI | Japan | JAPONICA |
| 68 R 5039 | United States | JAPONICA |
| AZ ROS 637 | United States | JAVANICA |
| AMBARBY WHITE | United States | JAPONICA |
| TianYouHuaZhan | China | INDICA |
| BAEK NA | Korea Rep | JAPONICA |
| BOOL DO | Korea Rep | JAPONICA |
| Ⅱ You 725 | China | INDICA |
| NIPON | Portugal | INTERMEDIATE (HYBRIDS) |
| BAEKSEOK | Korea Rep | JAPONICA |
| BANCHONJO | Korea Rep | JAPONICA |
| BIJARI | Korea Rep | JAPONICA |
| H 50(4) | Argentina | INDICA |
| H 68(9) | Argentina | INDICA |
| H 81(20) | Argentina | INDICA |
| JAPONESITO 3 MESES F.A. | Argentina | INDICA |
| YERUA PA | Argentina | JAVANICA |
| BEUMGANGBYEO | Korea Rep | INTERMEDIATE (HYBRIDS) |
| Luo Hong 4A | China | INDICA |
| ANBARBOO FARS | Iran | INDICA |
| BOHOTO BALOOCHESTAN | Iran | INDICA |
| DARBARI ROODBAR | Iran | INDICA |
| GREDEH FARS | Iran | INDICA |
| GREDEH KALATI | Iran | INDICA |
| GHALLEH GHASSEM | Iran | INDICA |
| CHUBU 38 | Japan | JAPONICA |
| CHUBU-MOCHI 37 | Japan | JAPONICA |
| BANATA 35 | Portugal | JAPONICA |
| ESTRELA A | Portugal | INDICA |
| BANDO | Korea Rep | INDICA |
| CHIKANARI 1 | Japan | INDICA |
| CHIYODA WASE | Japan | INDICA |
| AZO WASE | Japan | INDICA |
| CHIYONISHIKI(NORIN 276) | Japan | JAPONICA |
| ASUKAMINORI(NORIN 281) | Japan | JAPONICA |
| KAABY VILLAGE FAIYUM CITY | Egypt | Unclassified |
| SANURIS FAIYUM | Egypt | Unclassified |
| SLEMA | Egypt | Unclassified |
| BONNET BELL | Italy | INDICA |
| BELGIOIOSO | Italy | JAPONICA |
| CERVO | Italy | JAPONICA |
| DEDALO | Italy | INDICA |
| ICARO | Italy | INDICA |
| IDRA | Italy | INDICA |
| HASSANY | Iran | INDICA |
| ELBA | Italy | JAVANICA |
| EUROSE | Italy | INTERMEDIATE (HYBRIDS) |
| GRALDO | Italy | INDICA |
| BAYAZ CELTIK | Turkey | INDICA |
| DERVIS | Turkey | INDICA |
| KARAKILCIK | Turkey | INDICA |
| KIRMIZI CELTIK | Turkey | INDICA |
| MALATYA SARISI | Turkey | INDICA |
| MORAKI | Turkey | JAPONICA |
| SARIKILCIK | Turkey | JAPONICA |
| TIRE-82 | Turkey | INDICA |
| KAFR EL-DAUWAR | Egypt | Unclassified |
| GRITNA | Italy | INDICA |
| BORYEONG 3 | Korea Rep | Unclassified |
| AKCELTIK | Turkey | JAPONICA |
| H 305-84 | Hungary | Unclassified |
| ITALICA CAROLINA | Poland | JAPONICA |
| ARABI | Egypt | JAPONICA |
| ERYTHROCEROS HOKKAIDO | Poland | JAPONICA |
| SAB INI | Egypt | JAPONICA |
| SULTANI | Egypt | JAPONICA |
| ELVO::IRGC 82422-1 | Italy | JAPONICA |
| PI 282203::IRGC 16292-1 | Hungary | JAPONICA |
| Ⅱ YouHang 1 | China | INDICA |
| YongYou 12 | China | JAPONICA |
| YangLiangTou 6 | China | INDICA |
| ShanYou 63 | China | INDICA |
| LiangYouPei 9 | China | INDICA |
| LiangYou 6326 | China | INDICA |
| LiangYou 287 | China | INDICA |

Table S2. Generalized additive model (GAM) analysis results (the deviance explained and the corresponding *P* values of the smoothing term) for rhizosphere fungal taxonomic composition and specific plant functional traits.

| Plant functional trait | Fungal taxonomic composition | | | |
| --- | --- | --- | --- | --- |
|  | Rhizosphere | | Endosphere | |
|  | Deviance explained (%) | *P* value | Deviance explained (%) | *P* value |
| Root biomass | **17.2** | **0.006** | <0.01 | 0.744 |
| Shoot biomass | 3.43 | 0.140 | <0.01 | 0.983 |
| Whole plant biomass | **7.99** | **0.023** | <0.01 | 0.901 |
| Root: shoot biomass ratio | **10.9** | **0.045** | <0.01 | 0.946 |
| Root N content | **11.7** | **0.035** | 6.02 | 0.195 |
| Shoot N content | **13.4** | **0.004** | 2.04 | 0.259 |
| Root N accumulation | **15.9** | **0.011** | <0.01 | 0.761 |
| Shoot N accumulation | **10.1** | **0.013** | <0.01 | 0.655 |
| Whole plant N accumulation | **11.3** | **0.011** | <0.01 | 0.606 |

Table S3. Generalized additive model (GAM) analysis results (the deviance explained and the corresponding *P* values of the smoothing term) for rhizosphere fungal functional composition and specific plant functional traits.

| Plant functional trait | Fungal functional composition | | | |
| --- | --- | --- | --- | --- |
|  | Rhizosphere | | Endosphere | |
|  | Deviance explained (%) | *P* value | Deviance explained (%) | *P* value |
| Root biomass | **19.8** | **<0.001** | <0.01 | 0.997 |
| Shoot biomass | 0.47 | 0.458 | <0.01 | 0.525 |
| Whole plant biomass | **10.2** | **0.033** | <0.01 | 0.726 |
| Root: shoot biomass ratio | **12.1** | **0.004** | <0.01 | 0.635 |
| Root N content | **11.4** | **0.037** | **13.6** | **0.048** |
| Shoot N content | **13.9** | **0.011** | 7.32 | 0.113 |
| Root N accumulation | **11.5** | **0.005** | 0.49 | 0.334 |
| Shoot N accumulation | **10.7** | **0.030** | 4.45 | 0.097 |
| Whole plant N accumulation | **11.8** | **0.029** | 4.55 | 0.094 |
